# The profile of adolescent patients presenting to a tertiary maternal mental health clinic

**DOI:** 10.4102/sajpsychiatry.v29i0.2185

**Published:** 2023-12-20

**Authors:** Luzaan M. Cooke, Sanushka Moodley, Laila Paruk

**Affiliations:** 1 Department of Psychiatry, Faculty of Health Sciences, University of the Witwatersrand, Johannesburg, South Africa

**Keywords:** adolescent pregnancy, maternal mental health, major depressive disorder, adjustment disorder peripartum mental illness, substance use, education

## Abstract

**Background:**

Between March 2021 and April 2022, there were 90 037 documented adolescent pregnancies in South Africa. Statistics SA reports that this number is growing. Pregnancy places adolescents at greater risk of psychiatry-related morbidity and may have far-reaching consequences for their children. To date, there is no published data describing the patient profile of adolescent pregnancies in Gauteng Province, South Africa.

**Aim:**

To describe the patient profile (demographics, schooling history and type of accommodation), pregnancy-related factors, substance use habits and contraceptive use in pregnant adolescents seen at a tertiary care maternal mental health clinic (MMHC).

**Setting:**

The MMHC at Chris Hani Baragwanath Academic Hospital, Soweto, Johannesburg, South Africa.

**Methods:**

A retrospective file review of all pregnant adolescents referred to the MMHC between January and June 2022.

**Results:**

The mean age of the patients was 15.2 years; 72% attended school and 97.4% planned to return. Most pregnancies were unplanned (97.9%), wanted (84%) and presented in the second (41.24%) and third (51.55%) trimesters. Most did not report using substances (76.7%). Fifty percent of the participants met the criteria for a major depressive disorder. Repeating a grade was an identifiable risk factor for an increased Edinburgh depression score. An unplanned pregnancy was associated with a higher risk factor assessment.

**Conclusion:**

Pregnant adolescents represent a vulnerable population group. A greater understanding of this patient profile may inform early psychiatric and psychosocial interventions, improved service delivery and help-seeking behaviour.

**Contribution:**

This study gives significant insights into the challenges faced, as well as the health and social needs of pregnant adolescents. This contributes to wholistic care and opportunities for early intervention, including awareness of contraceptive use and the risks of substance use and adolescent pregnancy on mental health, benefiting all South African adolescents.

## Introduction

Adolescent pregnancies are a global concern, especially in disadvantaged communities and developing regions, where approximately 21 million adolescent girls aged 15–19 become pregnant annually.^1^ Sub-Saharan Africa has the highest population of children born to adolescents, with a rate of 143 births per 1000 adolescents aged 15–19 years.^2,3^ Among these, it is reported that 35% of pregnancies were unplanned and unwanted.^4^ In South Africa, there were 90 037 documented adolescent pregnancies between March 2021 and April 2022^5^, and this number continues to grow.^6^

Worldwide adolescent pregnancies occur more in disadvantaged communities where these pregnancies are thought to be driven by poor education and poverty.^1^ South Africa is classified as a low- to middle-income country, with a significant percentage of the population falling within a low socioeconomic status.^7^ In South Africa a key socio-economic indicator is the type of accommodation that a person or household resides in.^8^ A previous study done in Nigeria investigated the socio-demographic risk factors for unintended adolescent pregnancies, and found that the age of the adolescent, educational level and type of place of residence were statistically associated with adolescent pregnancy.^2^ Poor school performance has also been shown to be a strong marker for the increased likelihood of a pregnancy while enrolled in school, and of dropping out of school at the time of pregnancy.^9^ Poor school performance limits the likelihood that girls who experience a pregnancy will return to school.^9^ This lower level of education in turn has a negative effect on future employment.^10^

In South Africa, little is known about adolescent pregnancies, despite these pregnancies having far-reaching consequences for their children.^11^ Patel et al. found younger maternal age, poor social and family support and inadequate access to health and educational facilities place a greater risk of psychiatric-related morbidity on these adolescents.^12,13,14^ This may be explained using the diathesis-stress model, which describes the interaction between a person’s predisposition to mental illness and environmental stressors, such as adolescent pregnancy and the onset of psychopathology.^15^ The most common mental health disorders during this vulnerable period are anxiety and depression.^11^

Despite maternal mental illness being highly prevalent in low- and middle-income countries, it is often not adequately screened for detected or treated.^16^ Delay in detection and treatment has significant consequences extending through the antenatal and beyond the postpartum period.^16^ In South Africa, a qualitative study describing factors affecting the first 1000 days of infant life, which encompasses conception and the antenatal period, found that being pregnant as an adolescent causes significant stress for the adolescent, which in turn impacts the infant’s development.^17^ Growth and neurodevelopment are established in this period, making it a time of increased vulnerability that impacts the infants’ social, cognitive, physical and emotional development.^17,18,19^ A cohorts analysis, including South Africa’s Birth to 30 study cohort, has shown that younger mothers were more likely to have a child that is stunted at 2 years and who does not complete secondary school.^20^ Maternal anxiety and depression during this vulnerable antenatal period have been associated with an increased risk for neurodevelopmental disorders and poor emotional adjustment in their offspring.^21^ According to the DSM 5, neurodevelopmental disorders are defined as a group of conditions with onset in the developmental period, including deficits that produce impairments of function.^22^ Neurodevelopmental disorders comprise intellectual disability, communication disorders, autism spectrum disorder, attention-deficit/hyperactivity disorder, neurodevelopmental motor disorders and specific learning disorders.^22^

Contraception is an essential tool to prevent unwanted pregnancies. In developing regions, as many as 10 million pregnancies occurring annually among adolescents aged 15–19 are unplanned.^1,23^ A study across 31 countries (23 in sub-Saharan Africa) found a high unmet need for pregnancy prevention.^24^ Qualitative research regarding the perception of contraception services in South Africa revealed that adolescents aged 15–19 experience challenges accessing contraceptives on an interpersonal and health service level.^25^ Critical barriers identified were lack of support from parents or sexual partners; health-service barriers included the negative attitude of health care providers.^25^

Adolescence is often characterised by increased risk-taking behaviour.^26^ In South Africa, substance use is common in adolescence. In the 2002 and 2008 youth risk behaviour surveys, 20% – 22% of grade 8–11 adolescents used substances.^27^ Adolescents who use substances are likelier to engage in unprotected sexual practices that can lead to pregnancy.^28,29^ Substance use during pregnancy may cause significant complications for the expectant mother and the developing child.^30^ Substance use during pregnancy may lead to fatal complications.^31^ Substance use disorders in pregnant women have been associated with poorer compliance to antenatal care, thus putting both mother and infant at risk.^32^ In South Africa, it is reported that between 3.6% and 8.8% of pregnant women use illicit substances.^31^ The most commonly used substances in South Africa include cannabis and methamphetamine.^31^ The use of opioids is also on the rise.^31^ Cannabis use in pregnancy has been linked to preterm labour, low birthweight and small for gestational age infants.^31^ The developing brain is especially vulnerable in the prenatal period, where studies have linked prenatal exposure to marijuana to injury in the prefrontal region of the brain.^30^ Cocaine and methamphetamine use in pregnancy has been associated with maternal morbidity such as hypertensive crisis, myocardial infarction, cerebrovascular events, pulmonary oedema, aortic dissection, renal failure and postpartum haemorrhage.^31,32^ Foetal complications from cocaine and methamphetamine include preterm labour, lower birthweight, foetal loss, developmental and behavioural deficits.^31,32^ Opioid use increases the risk of third trimester bleeding and maternal death.

To date, there is no published data describing the patient profile of adolescent pregnancies, whose health and social needs differ from those of pregnant adult women,^33^ in Gauteng Province. This study aimed to describe the patient profile (including demographics, schooling history and type of accommodation), pregnancy-related factors, substance use habits and contraceptive use of pregnant adolescent patients seen at a tertiary care Maternal Mental Health Clinic (MMHC) in the hope of gaining a better understanding of the profile of adolescent pregnancies. This understanding may help the study populations clinicians to be more wholistic in their care as they may have a more in-depth understanding of the challenges these adolescents face. This study may also create the opportunity for early psychiatric and psychosocial interventions, improved service delivery and enhanced help-seeking behaviour, which would benefit all South African adolescents.

## Methods

### Study design

This retrospective study examined the records of all pregnant adolescent patients consulted at the outpatient MMHC at Chris Hani Baragwanath Academic Hospital (CHBAH) for 6 months from January to June 2022. Data were collected from July to December 2022. All data were collected anonymously. Demographic profiles, schooling history, type of accommodation, substance use habits, contraceptive use and pregnancy-related factors were collected. The presence of a diagnosed psychiatric illness and assessment scores from the RFA (Risk Factor Assessment) and EDS (Edinburgh Depression Scale) were documented. These scales were used to screen for depression in all patients.

### Setting

Chris Hani Baragwanath Academic Hospital is a tertiary academic hospital in Soweto, Johannesburg. It services a large catchment area in south Gauteng and includes acute inpatient adult, adolescent, child and outpatient psychiatric services. It receives referrals from district hospitals and specialised psychiatric clinics in its catchment area. The MMHCs at CHBAH and Rahima Moosa Mother and Child Hospital, are the only available specialised psychiatric services to assess pregnant adolescents, in the public sector for southern Gauteng. Referrals are received from the Departments of Obstetrics and Gynaecology and Psychiatry at CHBAH and local clinics in both disciplines. Referrals include pregnant women known to have mental illness, pregnant women who are screened as having an increased risk for mental illness, pregnant women presenting with acute mental illness and all pregnant adolescents under 18.

### Study population

All adolescent pregnancies aged 10–18 years referred to the MMHC at CHBAH over 6 months from January to June 2022 were included in the study. Sample size was determined using an assessment of Z scores and expected frequencies, taking the prevalence of adolescent pregnancies in Gauteng Province in 2021 at 8.9%, to obtain a sample size of 124 patients that will detect statistical significance at the fifth percentage level at a low effect size. At a medium effect size, statistical significance can be expected with a sample of 100 patients.

### Data collection

Data were collected using a retrospective file review, documenting assessments done by psychiatric medical officers, registrars and consultants. The information was collected and documented from patient files using a data sheet. Identifying data were not included in the data sheet, thus maintaining anonymity. The files for patients are kept in a locked filing system within the psychiatry department at CHBAH.

### Data analysis

The data for this study were collated in Microsoft Excel™. Statistical analyses were conducted in R software (version 4.00; www.R-project.org). Analyses were conducted on categorical variables only. Pearson’s chi-squared tests (goodness of fit) were used to assess whether the distribution of counts of socio-demographic, pregnancy conditions, substance use habits and diagnosis (psychiatric and comorbid) variables deviated from a null model. Binary post-hoc tests were used to assess pairwise comparisons of significant outcomes. The prevalence of psychiatric risk on the RFA and EDS scores was calculated as percentages of the total sample size with 95% CIs. Fisher’s exact tests were used to test the associations between selected socio-demographic/pregnancy variables and the RFA and EDS scores. Statistical tests were two-tailed, and the model significance was set at 0.05. Continuous variables are reported as mean and standard deviation, and categorical data are reported as percentages and presented in tables, text and charts.

### Ethical considerations

This study is a retrospective record review. All identifying data were omitted from the data sheet to maintain anonymity. Ethics approval was obtained from the Human Research Ethics Committee (Medical) of the University of the Witwatersrand (Clearance Certificate M220401) and the CEO of CHBAH.

## Results

Data from the 97 participants were analysed. Not all questions were recorded in the patient file, so the sample size reported varies by variable.

### Demographic profile

Participants had a mean age of 15.2 (standard deviation [s.d.] = 1.03), ranging from 13 to 18 years. Table 1 provides the socio-demographic characteristics of the participants. The majority of participants were in mainstream schooling, with three participants in remedial schooling (Table 1). Most participants were in Grade 9 or 10, and most did not repeat any grades. All but two participants planned to return to school. There was no difference in participants living in formal and informal dwellings, but this could be because of the low number of responses. The majority of participants lived with their families (Table 1). Three participants reported living with a partner, and one participant reported living alone.

**TABLE 1:** Socio-demographic variables of study participants.

Variables	Count	Percent	Statistics
**Schooling**			***p* < 0.001**
In school	67	72.0	-
Not in school	26	28.0	-
**Education school type**			***p* < 0.001**
Mainstream school	73	96.1	-
Remedial school	3	3.9	-
**Current grade**			***p* < 0.001**
6	1	1.1	-
7	2	2.1	-
8	8	8.5	-
9	26	27.7	-
10	39	41.5	-
11	17	18.1	-
12	1	1.1	-
**Number of times a grade was repeated**			***p* < 0.001**
None	65	87.5	-
1	9	12.5	-
**Education continuation plan**			***p* < 0.001**
No plan to return to school	2	2.6	-
Plan to return to school	74	97.4	-
**Type of accommodation**			***p* = 0.317**
Formal dwelling	6	66.7	-
Informal dwelling	3	33.5	-
**Type of accommodation – Occupants**			***p* < 0.001**
Living alone	1	1.1	-
Living with family	91	95.8	-
Living with partner	3	3.2	-

Count and percentage data are shown. Statistics = chi-squared tests per variable; significant outcomes are shown in bold.

### Pregnancy-related factors

Table 2 provides pregnancy-related characteristics of the participants. Most (97.9%) pregnancies were unplanned, and 89.4% of participants reported that the pregnancy was wanted. Significantly more participants were primigravida. No participants had a history of termination of pregnancy. Most participants were in the second and third trimesters of pregnancy when they visited the clinic (Table 2). Three participants mentioned using contraceptives, but the type of contraceptive used was not documented.

**TABLE 2:** Pregnancy-related variables of patients in the study.

Variables	Count	Percent	Statistics
**Pregnancy – Conception**			***p* < 0.001**
Planned	2	2.10	-
Unplanned	92	97.90	-
**Pregnancy – Decision**			***p* < 0.001**
Unwanted	10	10.60	-
Wanted	84	89.40	-
**Gravidity**			***p* < 0.001**
1	95	97.90	-
2	2	2.10	-
**Parity**			***p* < 0.001**
0	95	97.90	-
1	2	2.10	-
**History of TOP**			***p* < 0.001**
No	97	100.00	-
Yes	0	0.00	-
**Trimester of presentation at the clinic**			***p* < 0.001**
First trimester	7	7.22	-
Second trimester	40	41.24	-
Third trimester	50	51.55	-

TOP, Termination of Pregnancy.

Count and percentage data are shown. Statistics = chi-squared tests per variable; all were significant.

### Substance use habits

Table 3 provides the substance use information for patients in the study. Most (76.7%) participants reported no substance use. Of those using substances, more participants were not using substances during the study period (Table 3).

**TABLE 3:** Substance use in participants in the study.

Variables	Count	Per cent	Statistics
**History of substance use**			***p* < 0.001**
No	66	76.7	-
Yes	20	23.3	-
**Current use of substances**			***p* < 0.001**
No	18	94.7	-
Yes	1	5.3	-

Count and percentage data are shown. Statistics = chi-squared tests per variable; all were significant.

Most participants reported drinking alcohol (*n* = 19), three reported smoking cannabis and one smoked tobacco. None of the participants reported using methamphetamines, amphetamines, opioids, benzodiazepines, cocaine or nyaope. Five participants (33%) reported drinking alcohol weekly, and 10 (67%) did not provide any information. Three participants (50%) drank alcohol for less than a year, two (33%) for 1–5 years, and one (17%) for more than 5 years. No further details were available for participants using cannabis and tobacco. Eleven participants (100%) reported not having withdrawal symptoms at the time of assessment.

### Psychiatric diagnosis and risk profile

A prevalence of high patient risk (i.e. a score ≥ 3) was 44.4% (95% LCI (Lower Confidence Interval): 0.30; UCI (Upper Confidence Interval): 0.59; *n* = 45) from the risk factor assessment scores. Using the Edinburgh Depression Scale score, the prevalence of high patient risk (i.e. a score ≥ 13) was 47.7% (95% LCI: 0.36; UCI: 0.60; *n* = 34).

Thirty respondents answered questions for the Risk Factor Assessment and Edinburgh Depression Scales. Of these, 14 (47%) had a high risk (≥ 3 and ≥ 13) on both scoring systems.

Table 4 shows the psychiatric and comorbid diagnoses in the participants. Significantly more participants had major depressive disorder (52.6%), followed by adjustment disorder (21.1%). There were five other disorders and two comorbidities (Table 4).

**TABLE 4:** Diagnosis (psychiatric and comorbid) in the study.

Variables	Count	Per cent	Statistics
Major depressive disorder	20	52.6	***p* < 0.001**
Adjustment disorder	8	21.1	
Cannabis use disorder	2	5.3	
Epilepsy (comorbid)	2	5.3	
Post traumatic stress disorder	2	5.3	
Dysthymia	1	2.6	
Borderline personality disorder	1	2.6	
Intellectual disability mild	1	2.6	
Rvd (comorbid)	1	2.6	

Count and percentage data are shown. Statistics = chi-squared test: the outcome was statistically significant.

**FIGURE 1:**
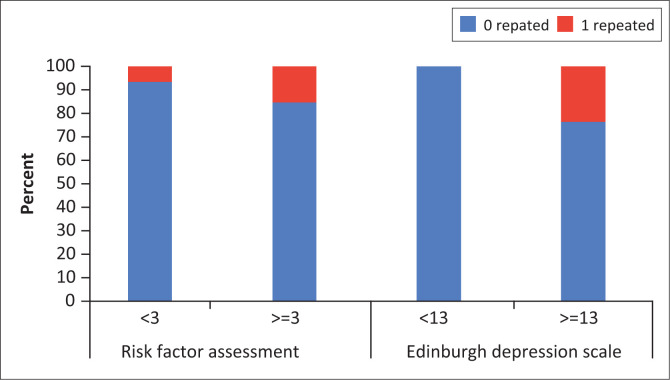
The relationship between grades repeated and low and high-risk factors in the Risk Factor Assessment < 3 = low; ≥ 3 = high) and Edinburgh Depression Scale (< 13 = low; ≥ 13 = high).

**FIGURE 2:**
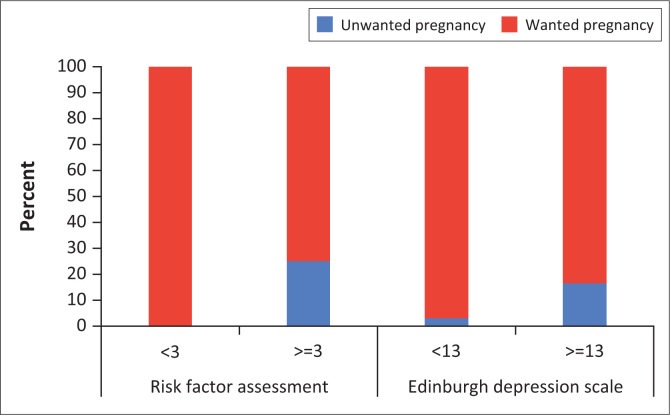
The relationship between whether the pregnancy was wanted or unwanted and low and high-risk factors in the Risk Factor Assessment (< 3 = low; ≥ 3 = high) and Edinburgh Depression Scale (< 13 = low; ≥ 13 = high).

The association of two variables with the Risk Factor Assessment and Edinburgh Depression Scale assessments is shown in Figure 1 and Figure 2. For the number of grades repeated, a significantly greater proportion (*n* = 5; 24%) of participants who repeated a grade also had a high-risk profile on the Edinburgh Depression Scale (Fisher’s exact test: *p* = 0.048) versus no participants who repeated a grade and had low risk on this scale (Figure 1). There was no significant association between grades repeated and Risk Factor Assessment (Fisher’s exact test: *p* = 0.583). For wanted and unwanted pregnancies, a significantly greater proportion (*n* = 5; 25%) of participants with an unwanted pregnancy had a high-risk profile on the Risk Factor Assessment scale (Fisher’s exact test: *p* = 0.016) versus participants who did not want to keep the pregnancy and had a low risk for this scale (Figure 2). There was no significant association between wanting to keep the pregnancy or not and Edinburgh Depression Scale (Fisher’s exact test: *p* = 0.091).

## Discussion

This study describes the patient profile, pregnancy-related factors and substance use habits of 97 participants seen at the outpatient MMHC at CHBAH from January to June 2022.

### Demographics

The mean age of patients presenting to the clinic was 15.2 years, ranging from 13 to 18 years. This is in keeping with the World Health Organization statistics that most adolescent pregnancies worldwide (about 16 million) are between 15 and 19 years. Fewer adolescent pregnancies (about 1 million) are younger than 15 years.^34^ Reasons for this could include that, despite older adolescents being able to negotiate safer sexual practices with their partners and having more knowledge of contraceptives, they still face barriers in accessing and using contraceptives.^35^ Adolescents are an at-risk group for developing psychiatric-related morbidity, as most mental illnesses start in adolescence and early adulthood.^36^ Adolescents are also at higher risk of using substances.^27^ The National Mental Health Policy Framework (NMHPF) and Strategic Plan for 2023–2030 highlight the importance of developing mental health promotion and prevention programmes for critical developmental life stages such as pregnancy and adolescence.^37^ Adolescents are considered a special population by the NMHPF due to most mental health disorders originating in childhood and adolescence, with roughly 50% of mental disorders presenting by the age of 14.^37^ The policy also emphasises that, in South Africa, childhood adversity, which includes all forms of abuse, has been associated with mood and substance use disorders, which increases the chances of children not completing school.^37^ Despite being a vulnerable population, services for adolescent mental health are severely lacking and have been identified in the NMHPF as an area where intervention is needed^37^. It has been proposed that all district health services routinely screen for mental illness during pregnancy and that adolescent mental health disorders are detected in primary health care clinics and referred where appropriate.^37^

### Schooling

A common concern for pregnant adolescents is the ability to further their education.^38^ The educational level prior to pregnancy has been linked with better health and well-being, which could possibly compensate for the mother’s age and the child’s IQ later in life.^38^ We found that 72% of pregnant adolescents were still attending school, and 97.4% planned to return. This contrasts with the data reported by Statistics South Africa in 2016, which showed only 51.5% of adolescent mothers attended school.^39^ This significant difference in statistics could be because, although pregnant adolescents are motivated to continue their schooling, it might not be possible practically. Previous studies done in South Africa showed that poverty and unmet childcare needs are major contributors preventing adolescents from returning to school postpartum.^38^ A qualitative study conducted in Soweto, South Africa, showed that there are 2 pathways for pregnant adolescents while still enrolled in school: some managed to return to school, delaying their next pregnancy by 5–10 years and those who did not return to school and went on to have more children.^20,40^ This in turn has a negative impact on future work opportunities and careers, contributing to unemployment among the youth.^10^ Supporting adolescents to return to school after childbirth is paramount, as prolonged absence increases the risk of not returning.^38^ Further research is needed to compare the rates of pregnant adolescents planning to return to school, the rates of re-enrolment postpartum and other barriers preventing re-enrolment.

Data reporting on adolescent pregnancies occurring in mainstream schools versus remedial schools in South Africa is scarce. This study found that most adolescents attended a mainstream school (96.1%), and only 3.9% attended a remedial school. Most patients had never repeated a grade (87.5%). This may be a protective factor for these patients as an appropriate age for grade before pregnancy has been associated with greater odds of school return.^38^ For the number of grades previously repeated, a more significant proportion (24%) of patients that repeated a grade also had a high-risk profile on the Edinburgh Depression Scale. This high percentage of pregnant adolescents never repeating a grade contrast with research done in KwaZulu-Natal, where non-pregnancy-related grade repetition was strongly associated with becoming pregnant while still enrolled at school and not returning following a pregnancy-related absence.^41^

### Accommodation

This study found that most adolescent patients resided with their families (95.8%). This echoes previous research showing that most pregnant adolescents still depend on their families for their place of residence, financial and schooling needs and emotional support during this time.^42^ Pregnant adolescents often experience psychological distress in disclosing the pregnancy and the biological father’s identity to the family fearing sanctions,^43^ a lack of support and stigmatisation.^10^ The parents actual or anticipated reaction to the pregnancy may be a major factor in whether the adolescent keeps the baby or decides on abortion.^43^ After giving birth, these adolescents need family support in caring for the new-born baby, as a major contributing factor for an adolescent mother to continue with schooling is the support of her family, especially the support of her mother in assisting with childcare.^10^ Evidence on the burden of care experienced by these families indicates high levels of financial and emotional stress.^44^ Further research is needed into the burden of care for the families of pregnant adolescents to inform practice on the support needed.

Too little data were captured on the type of accommodation to make statistical associations.

### Pregnancy-related factors

Most adolescent pregnancies were unplanned (97.9%) although most were wanted (89.4%). This contrasts with a study in sub-Saharan Africa, where 35% of adolescent pregnancies were unplanned and unwanted.^4^ A possible reason for this discrepancy could be that most unwanted pregnancies end up in early termination, and these adolescents were not seen at the study facility. In the United States, 91% of all pregnancies in 15- to 17-year-olds are unplanned,^45^ which correlates with our findings. When comparing wanted and unwanted pregnancies, a significantly bigger proportion (25%) of patients who did not want the pregnancy had a high-risk profile on the Risk Factor Assessment scale. This was also shown in the UK, where unplanned pregnancy and negative feelings about the pregnancy resulted in significant psychological distress.^46^

Most teenagers said this was their first pregnancy (97.9%). Two participants reported having a previous pregnancy. Among the two participants that reported a previous pregnancy, none reported a previous termination. This differs significantly from a study conducted in Soweto, South Africa, reporting that 23% of pregnancies in 13- to 16-year-olds and 14.9% in the 17- to 19-year age group resulted in abortion.^3,4^ A possible reason for this significant difference in reported previous terminations could be that the patients in our study were reluctant to disclose previous pregnancies that ended with a termination due to the associated stigma or that those who terminate the pregnancy never reach the MMHC. Most patients presented to the MMHC in the second (41.24%) and third trimester of pregnancy (51.55%). Very few patients were seen during their first trimester (7.22%) when termination of pregnancy is legal in South Africa on patient request and without medical indication.^47^ Further investigation is needed to ascertain why adolescents only present in the second and third trimesters, specifically examining the timeline from initial presentation to primary health care clinics to being seen at the MMHC. Earlier presentation to the MMHC can allow mental health problems to be addressed timeously, resulting in an improved outcome for both mother and baby.

### Substance use habits

Significantly more patients reported no substance use (76.7%). Of those using substances, most did not use them during the study period (94.7%). This differs significantly from the United States, where pregnant adolescents reported greater drug use than their same-age non-pregnant peers (13.8%).^45^ In South Africa, 20% – 22% of Grade 8–11 adolescents reported using substances in the 2002 and 2008 youth risk behaviour survey.^27,48^ In the same survey, the percentage of learners who reported ever having used dagga was 12.8%, inhalants 11.1%, Mandrax 6.0%, cocaine 6.4%, heroin 11.5%, club drugs 5.8% and over-the-counter or prescription drugs 15.5%.^27,48^ The prevalence of alcohol use among adolescents ranges from 22 to 53.8%.^49^ This discrepancy could be accounted for if there was under-reporting of substance use by the study population. Another explanation could be the difference in setting where the surveys were conducted, in a health care setting versus in the community, for example, at a school, where the adolescents may feel less stigmatised when reporting substance use. Early substance use intervention would benefit adolescents, and the NMHPF and strategic plan for 2023–2030 highlight the need for coordinated services between the Departments of Health and Social Development to provide prevention measures and treat substance use disorders.^37^

### Risk factor assessment and Edinburgh Depression Scale

A prevalence of high patient risk (i.e. a score ≥ 3) from the Risk Factor Assessment scores was 44.4%. Using the Edinburgh Depression Scale score, the prevalence of high patient risk (i.e. a score ≥ 13) was 47.7%. Thirty respondents answered questions for both the Risk Factor Assessment and Edinburgh Depression Scale; of these, 14 (47%) had a high risk (≥ 3 and ≥ 13) on both scoring systems. This agrees with several studies suggesting that adolescent mothers are at higher risk of developing depression.^50^ This high risk can significantly impact the adolescent mother and the unborn child. Antenatal maternal depression has been associated with an increased risk of neurodevelopmental disorders and poor emotional adjustment in their offspring.^21^ Maternal depression can lead to adverse birth outcomes such as low infant birth weight, being small for gestational age and premature birth.^51^ Undiagnosed and untreated prenatal depression can continue as postpartum depression.^52^ Non-systematic reviews have identified that children born to mothers with untreated postpartum depression are at risk for poor cognitive function, behavioural inhibition, emotional maladjustment and externalising disorders.^53^ A mother with untreated postpartum depression is likelier to use alcohol or illicit substances and have breastfeeding and relationship problems.^53^

### Diagnosis

Significantly more patients had major depressive disorder (52.6%), followed by adjustment disorder (21.1%). There were five other disorders and two comorbidities. There is a slightly higher incidence of Major Depressive Disorder among pregnant adolescents at the MMHC at CHBAH compared to the findings reported by Stacy C. Hodgkinson et al. in the study ‘Depressive Symptoms and Birth Outcomes among Pregnant Teenagers’ (16% – 44%).^54^ Further investigation would need to be done to ascertain the reasons for this difference.

### Contraception

Adolescent pregnancy prevention is an essential strategy for improving maternal and infant outcomes.^55^ A lack of access or knowledge of contraceptive use leads to early unwanted pregnancies.^55^ Unfortunately, no information on contraceptive use was recorded in patient files. This could speak to missed social interventions and understanding needs, given that 97.9% of pregnancies were unplanned. Prenatal contraceptive counselling is a topic that pregnant adolescents need to be educated about, as antenatal contraceptive counselling has been shown to increase postpartum contraceptive use by adolescents, thus preventing another unplanned pregnancy.^56^

### Limitations

In this study, retrospective file review methodology was employed. Firstly, this meant that the researcher was limited by the information that was present and recorded in the study population’s files. For example, there was limited information provided with regard to the adolescent’s living conditions, extent of family support and contraceptive use. Further research that includes self-report questionnaires specifically asking about contraception use and knowledge will be informative to help develop strategies to prevent adolescent pregnancies. Secondly, the patient assessments were done by a combination of psychiatric medical officers and registrars with different levels of skill and expertise. The assessments of the patient’s mental health were therefore not done on a standard level of expertise. Thirdly, the study took place over a short period of only 6 months, and a more extended study with a larger study population would improve the reliability of the results. Fourthly, the study took place at a single centre, and included only patients referred to the MMHC (a tertiary institution); therefore, the results cannot be generalised to other adolescent populations. A study involving multiple centres, at all levels of care, would be of benefit to generalise the findings.

## Conclusion

This review emphasises the patient profile of pregnant adolescent patients presenting to a tertiary maternal mental health facility. A greater understanding of this population’s health and social needs may inform early psychiatric and psychosocial interventions and improve service delivery and help-seeking behaviour. Collaboration between the Departments of Health, Social Development and Education is needed to further psychoeducation on contraceptive use, the adverse effects of substance use and how to screen for mental illness in pregnancy. The development of a standard format of assessments of pregnant adolescents would be helpful to both clinicians and primary-level healthcare workers. This will assist in the identification of those at greater risk, early identification of mental health concerns and intervention as pregnancy places adolescents at a greater risk of psychiatric-related morbidity, which could have far-reaching consequences for their children. Collaboration is also needed to facilitate the treatment of this vulnerable population’s substance use disorders. It is suggested that further research be conducted into the use of contraceptives in this population, as this data was not available for the study and could assist in preventing further adolescent pregnancies. Further research focussing on rates of school re-enrolment after childbirth and barriers preventing re-enrolment is needed, as this could help inform how these adolescents can be better supported to further their education.
